# Electronic cigarette or vaping use among adolescents in the United States: A call for research and legislative action

**DOI:** 10.3389/fpubh.2022.1088032

**Published:** 2022-12-01

**Authors:** Bretton Gilmore, Kelly Reveles, Christopher R. Frei

**Affiliations:** ^1^Pharmacotherapy Division, College of Pharmacy, The University of Texas at Austin, Austin, TX, United States; ^2^Pharmacotherapy Education and Research Center, School of Medicine, The University of Texas Health Science Center at San Antonio, San Antonio, TX, United States

**Keywords:** vaping, electronic cigarettes, adolescents, policy, United States, legislation

## Abstract

Vaping among adolescents is increasingly common and may result in poor health outcomes; however, little research has been conducted evaluating the risks of vaping among adolescents and the knowledge and perceptions that drive use. We must gain a better understanding of vaping outcomes and adolescents' perceptions while identifying potential ways to lessen or eradicate the health burdens associated with vaping. This knowledge could then inform robust educational and public health programs to prevent and mitigate vaping among youths. Health education incorporating a target populations' world view, spheres of influence, readiness, motivation, intention, and determination promotes informed decision making. There are few resources currently being allocated to the problem even though legislators and enforcement agencies are aware. We cannot simply rely on existing laws to serve as a sufficient deterrent to prevent underaged usage. Further efforts are needed in the areas of behavioral science, health education, and public policy to tackle this urgent public health concern.

## Introduction

Smoking is the number one preventable cause of death in the United States (US) (1). As an alternative to smoking, vaping was introduced in 1927, (2, 3) and ultimately became commercially available in the US in 2007. Electronic cigarettes come in a variety of devices including cigarette-like pens, pipes, hookahs, and tanks to emulate a smoking-type experience (4). They employ a battery to fuel a heating element to produce a vaper by atomizing liquid containing nicotine or tetrahydrocannabinol (THC) (5, 6) commonly referred to as juice, to a point where it aerosolizes before inhalation (7). These chemicals can sensitize the pleasure system and cause dependence (8).

Adolescents, who have never smoked traditional cigarettes, have shown a propensity to vape at young ages. Despite federal and state age restrictions for legal purchase of e-cigarettes or vaping products, it's estimated that 4.7 million youth vape (9). Approximately one-quarter of high school students reported vaping within the past 30 days (10). This may be partially due lack of health education programming and limited adherence by vape shops to verify customer age. Prior studies have documented vape shop ID check compliance at only 33% and 25% for entrance to establishment and point of sale, respectively (11).

Adolescents primarily express the reasons they begin vaping are due to curiosity, pleasure, and taste (11). Many adolescents and young adults experiment with the multitude of flavors with perceptions of harm shown to be directly tied to fruity or candied flavors as less harmful than tobacco flavors (12, 13). This trend suggests adolescents may not view vaping as traditional tobacco use. Furthermore, the flavors themselves have the potential to introduce additional toxins not seen in flavorless juice variations (14).

## Potential dangers of vaping among adolescents

Vaping has been associated with a higher risk of respiratory (e.g., cough 42%, shortness of breath 14%, chest tightness 6%), (15) circulatory (e.g., stroke 71%, 59% myocardial infarction, 40% heart disease), (16) and nervous system (e.g., headache 25%, dizziness 32%, taste changes 4%) (15) problems at alarming rates with e-cigarette, or vaping, product use-associated lung injury (EVALI) garnering much attention recently. While prior studies have documented acute airway and mucosal irritation, inflammation, and hypersecretion which can lead to hypoxemia and tachypnea in those who vape (17, 18) a study among adolescents failed to demonstrate a relationship between vaping and self-reported respiratory wheezing among adolescents when controlling for ethnicity, combustible tobacco exposure, and household knowledge, attitudes, and beliefs expressed as rules (19). Next, adolescents are often unaware of the addiction potential of nicotine and fail to draw a significant correlation between vaping and nicotine consumption (20, 21). Even infrequent use can lead to nicotine addiction, which has been shown to affect brain development (22, 23). Furthermore, oxidative stress resulting from vaping can lead to social maladjustment, learning deficits, poor impulse control, sleep disruption, depression, and even suicidal ideations (24). Vapers have a higher prevalence of depression and suicidality than nonusers (25, 26). Furthermore, adolescent brain development is extremely active during the formative years during middle and high school and adverse consequence of stunted cognitive maturation can lead to behavioral impairment, memory disruption, attention deficit, and decreased motor function and has been observed with prolonged vape use (27).

Despite these findings, the long-term effects of vaping, especially in adolescents, has yet to be robustly studied and requires significant investigation before all of the potential harms can be determined (28). Vaping is a relatively new mechanism of nicotine consumption which may not have existed temporally with sufficient time to manifest the most severe potential health outcomes (e.g., cancer).

## Discussion: A call for research and legislative action

We must gain a better understanding of vaping outcomes and adolescents' perceptions while identifying potential ways to lessen or eradicate the health burdens associated with vaping. Merely informing adolescents about safety concerns is doing little to discourage use; (29) therefore, more research is needed to understand their perceptions and risk tolerance for vaping. These findings could then inform robust educational and public health programs to prevent and mitigate vaping among youths.

Health education incorporating a target populations' world view, spheres of influence, readiness, motivation, intention, and determination promotes informed decision making. Informed decision making can then promote healthy behaviors and help prevent negative health outcomes. Positive health behaviors also serve as a reciprocal example for others influencing and reinforcing knowledge, attitudes, and beliefs complementary to social norming and reduction of negative peer pressure. Dissemination of information influences not only the individual in a target population but also the social culture in an organizational context. Adolescents often feel invincible and impervious to injury or disease. Their points of view are paramount whether assessing needs, planning interventions, or evaluating effectiveness of programs if we hope to remove barriers and encourage sound judgment. Studies have shown that influence that originates in the home is particularly effective at molding attitudes and beliefs in adolescents (30–32).

Next, a strategy to combat underage sale and distribution of vaping equipment and juice, should be pursued though policy analysis, agenda setting, formation, adoption, enactment, assessment, modification, and enforcement of current and future laws. This strategy should also prevent misinformation regarding vaping as a safe alternative to tobacco product use. Vape shops are exploiting health disparities of poorer inner-city communities and minorities at a disproportionate rate when compared to white, non-Hispanics rural areas. While health policies can be reflected in executive orders, laws, ordinances, position statements, regulations, and formal/informal rules, a common theme, regardless of final format or version, is they all require implementation planning and enforcement. Foundational to such planning is the knowledge, attitudes, and beliefs of priority population if advocates and educators hope to tailor specific interventions.

There are few resources currently being allocated to the problem even though legislators and enforcement agencies are aware. We cannot simply rely on existing laws to serve as a sufficient deterrent to prevent underaged usage. In summary, vaping among adolescents is increasingly common and may result in poor health outcomes; however, little research has been conducted evaluating the risks of vaping among adolescents and the knowledge and perceptions that drive use. Further efforts are needed in the areas of behavioral science, health education, and public policy to tackle this urgent public health concern.

## Summary

Observational cohort studies are needed, especially in underrepresented and at-risk adolescent groups, to determine knowledge, perceptions, and health risks associated with vaping. Prospective studies are needed to identify interventions that effectively prevent and mitigate vaping among adolescents. This knowledge could then inform robust educational and public health programs to prevent and mitigate vaping among youths.

## Data availability statement

The original contributions presented in the study are included in the article/supplementary material, further inquiries can be directed to the corresponding authors.

## Author contributions

BG and CF: study concept and design and drafting of the manuscript. BG, CF, and KR: critical revision of the manuscript for important intellectual content. CF: study supervision. All authors contributed to the article and approved the submitted version.

## Conflict of interest

The authors declare that the research was conducted in the absence of any commercial or financial relationships that could be construed as a potential conflict of interest.

## Publisher's note

All claims expressed in this article are solely those of the authors and do not necessarily represent those of their affiliated organizations, or those of the publisher, the editors and the reviewers. Any product that may be evaluated in this article, or claim that may be made by its manufacturer, is not guaranteed or endorsed by the publisher.
